# Deformation and Cracking Resistance of MgO-Incorporated Cementitious Material: A Review

**DOI:** 10.3390/ma16020500

**Published:** 2023-01-04

**Authors:** Jinyuan Lu, Pan Feng, Hua Li, Qian Tian

**Affiliations:** 1 School of Material Sciences and Engineering, Southeast University, Nanjing 211189, China; 2 Jiangsu Key Laboratory of Construction Materials, Southeast University, Nanjing 211189, China; 3 State Key Laboratory of High Performance Civil Engineering Materials, Nanjing 210008, China

**Keywords:** MgO expansive agent, autogenous deformation, cracking resistance, deformation mechanism, cement additive, expansive concrete

## Abstract

In China, MgO-based expansive agent (MEA) has been used for concrete shrinkage compensation and cracking control for over 40 years. The expansive behavior of MEA in cementitious materials could be manipulated to some extent by adjusting the calcination process of MEA and influenced by the restraint condition of the matrix. It is key to investigate the factors related to deformation and cracking resistance so that the desired performance of MEA in certain concrete structures could be achieved. This paper reviews the influence of key parameters such as hydration reactivity, dosage, and calcination conditions of MEA, the water-to-binder ratio, supplementary cementitious material, aggregates, and curing conditions on the deformation and cracking resistivity of cement paste, mortar, and concrete with an MEA addition. The numerical simulation methods and deformation prediction models are then summarized and analyzed for more reasonable estimations.

## 1. Introduction

In the past 50 years, magnesium oxide has been applied as an expansive agent for concrete shrinkage compensation in massive civil and infrastructure projects in China and other countries [1]. The shrinkage deformation of concrete could arise from hydration heat, self-desiccation, loss of water to the environment, and other conditions [2,3,4]. Then, stress develops to some extent and induces cracking in concrete, which severely reduces the performance and durability of the structure. In order to mitigate shrinkage-induced cracking, one of the effective methods is expansive agents, which hydrate to products with increased volume or generate crystallization pressure during the growth of products to cause expansive deformation [5,6,7]. Among the existing expansive agents, magnesium oxide shows unique characteristics, including variable expansive behavior through the calcination procedure design, temperature sensitivity, and stable products [8,9,10]. These characteristics ensure the effectiveness of magnesium oxide to mitigate shrinkage deformation and its capability to cope with different conditions. Therefore, research has been focused on the factors related to the deformation and cracking resistance of magnesium-oxide-incorporated cementitious materials so that shrinkage deformation and cracking could both be minimized.

The factors involved in the deformation and cracking resistance could be classified into three categories: magnesium-oxide-related, cement-related, and curing-condition-related. As for magnesium-oxide-related factors, the dosage and reactivity of MgO are the two main factors that have received much attention and interest. The calcination procedure of magnesium oxide could be represented to some extent by the reactivity of MgO when studying its effect on deformation; however, some research shows insufficient reactivity acquired by using the neutralization method when the sample is a mixture of MgO from varied sources [11]. The influence of the calcination procedure could also be interpreted as the effect of physical properties of MgO, such as specific surface area, particle size distribution, and crystallite size [5], that could be altered by the calcination process. In addition, properties such as specific surface area and particle size are adjustable by the selection of raw material (magnesite or other minerals) or milling. Aside from the physical properties of MgO, the cementitious materials and aggregates have an impact on the expansive behavior of MgO. The water-to-binder ratio (w/b ratio), phase composition of cement, category, and content of supplementary cementitious materials are the main factors in MgO expansive behavior through affecting the hydration process of MgO, heat release, and the stiffness of the matrix [12,13]. Additionally, the influence of aggregates is mainly studied in terms of water adsorption and gradation. Furthermore, the curing humidity and temperature are essential for studies on the deformation of MgO-incorporated cementitious materials due to the temperature sensitivity of MgO and the significant promotion of expansion under water curing conditions [14,15]. To use MgO as an expansive agent for shrinkage compensation and cracking control in a variety of structures and conditions, it is necessary to summarize and analyze the factors influencing the expansive behavior of MgO, as well as the mechanism underlying this behavior.

This paper presents a literature review of the deformation and cracking resistance of MgO-incorporated cementitious materials by analyzing the related factors from three aspects: MgO properties, cement and aggregate, and curing conditions. The relationships between these factors and the deformation or cracking resistance are discussed to determine the possible principles and mechanisms. Meanwhile, the methods and models for the estimation of MgO-incorporated concrete (or paste) deformation are reviewed for understanding the driving force of expansion, which can act as a reference for the application of MgO in novel cementitious systems. Finally, several conclusions are drawn from the analysis, and the discussion reflects the scientific research progress on the deformation and cracking resistance of MgO-incorporated cementitious materials.

## 2. Deformation of Cementitious Materials with the Addition of MgO

### 2.1. Effects of Characteristics and Content of MgO

Magnesium oxide used as an expansive agent in cementitious materials is usually obtained from the calcination of minerals such as magnesite and dolomite that are rich in magnesium carbonate content, with a calcination temperature in the range of 700–1100 °C, and residence time from 0.5 to 2 h. The physical properties of MgO such as particle size distribution, specific surface area, and crystallite size are affected by the calcination temperature, residence time, and cooling rate of the calcination process. With elevated calcination temperatures or prolonged residence time, both the particle size and crystallite size of MgO increase, and the specific surface area of MgO decreases, leading to a reduction in defects [5,11,16,17]. Compared with air cooling, MgO cooling in a furnace significantly reduces reactivity by 50–130 s of reaction time [17]. To characterize the reactivity of MgO, a neutralization method is introduced, in which 1.70 ± 0.1 g of MgO is mixed with 200 mL citric acid solution with a concentration of 0.07 mol/L at 30 ± 1 °C, and the time when the indicator phenolphthalein in solution turns red is recorded as the reaction time of the sample [5,11,16,17]. Some research points out a limitation of the neutralization method in that it only reflects the relative reactivity under set conditions and does not quantitatively correspond with the expansion in cementitious materials. An ethylic acid buffer solution method and hydration heat evolution are proposed to evaluate the reactivity of MgO [16,18]. The reaction time is closely related to the specific surface area and the dissolution rate of MgO particles, which correlates to the hydration of MgO with its expansion behavior [5].

**Figure 1:**
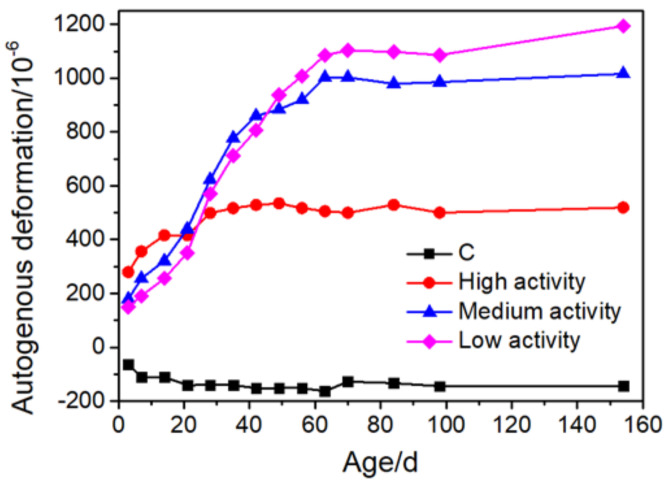
The influence of MgO reactivity on autogenous deformation of mortar (adapted from Ref. [23], 2019, Elsevier).

**Figure 2:**
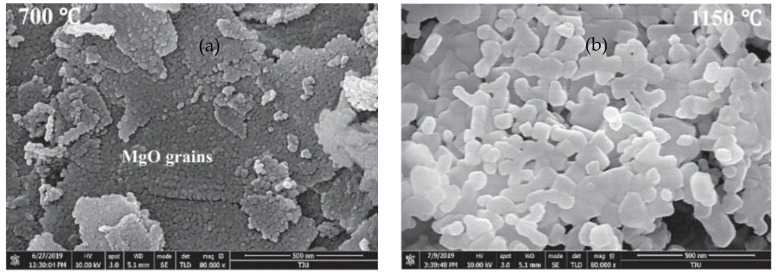
Microstructure comparison of MgO with different reactivity: (**a**) MgO with high reactivity; (**b**) MgO with low reactivity (reprinted with permission from Ref. [16], 2020, Elsevier).

**Figure 3:**
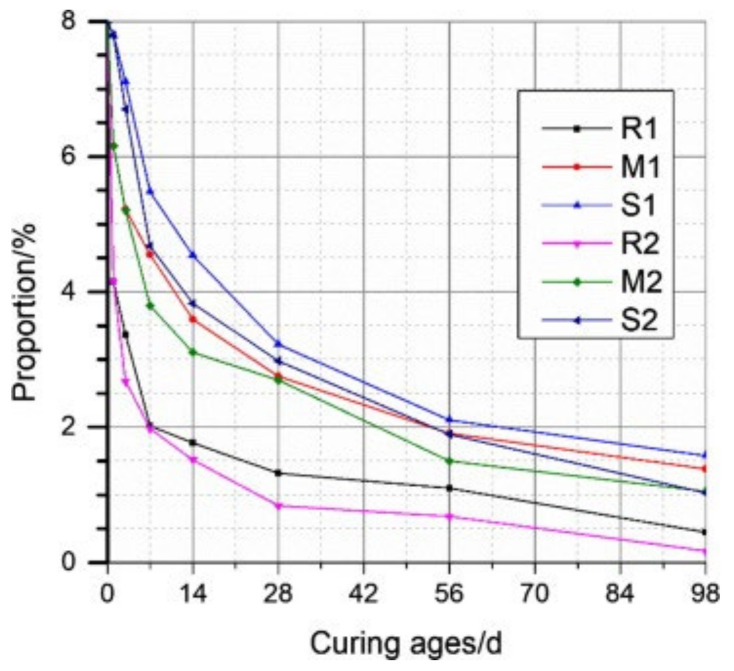
The development of periclase content during the hydration of cement paste with MgO characterized by QXRD (reaction times: R-60s, M-120s, and S-220s) (reprinted with permission from Ref. [23], 2019, Elsevier).

At different ages, the deformation of cementitious materials containing MgO of various reactivity shows different features. As shown in Figure 1, for mortar with high-activity MgO, the expansion under sealed conditions develops faster than that of mortar with low-activity MgO within 28 days and gradually reaches a constant expansive strain afterward. However, for mortar with low-activity MgO, the expansion grows slowly but continuously and exceeds that of mortar with high-activity and medium-activity MgO after about 60 d. With the increase in reactivity, the expansion rate rises, and the total expansive strain at later ages declines. For concrete, deformation develops in a similar pattern but is usually lower than that of mortar with the same w/b ratio [19,20,21,22]. Furthermore, elevated temperature and water curing can promote expansive deformation and accelerate the time when the deformation of low-activity MgO specimens exceeds that of high-activity MgO specimens [23,24]. As there are several methods for deformation characterization, which include strain gauges for sealed cylindrical concrete [19,25], corrugated tube method [17], and the comparator method [11,18,23], the comparability of the strain of MgO-incorporated cementitious materials acquired via different methods remains unresolved. The difference in the expansive behavior of MgO with different reactivity arises from the microstructure and hydration process of MgO particles. As shown in Figure 2a, for MgO with high reactivity, the particle is more porous, with smaller micrograins aggregating together. The specific surface area is higher, which leads to a faster dissolution rate during hydration, as shown in Figure 3. In Figure 2b, MgO particles with low reactivity have denser microstructure, larger grain sizes, and a less specific surface area, resulting in a slower dissolution rate [16]. This helps to clarify the high expansion strain and expansive rate of cementitious materials with high-activity MgO. Moreover, the mobility of the magnesium cation is limited during the hydration of MgO, causing the precipitation of brucite on the surface of MgO. The porous structure of MgO with high reactivity provides sites for nucleation and growth of brucite in interconnected pores inside particles, which limits the expansion [12].

**Figure 4:**
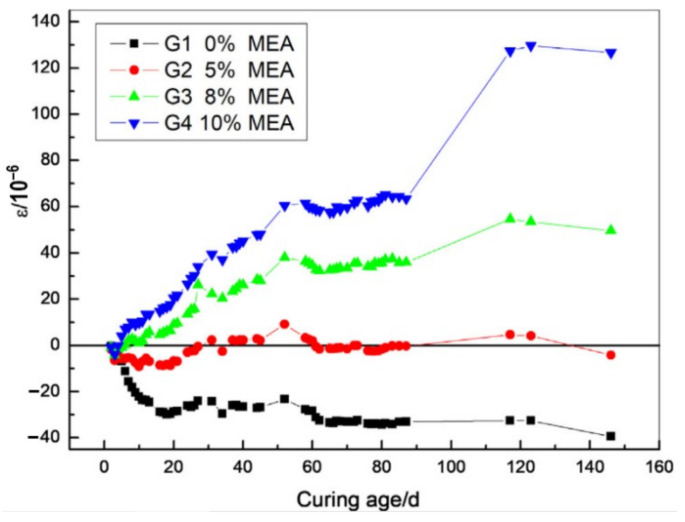
The influence of MgO content on the autogenous deformation of concrete (reprinted with permission from Ref. [26], 2019, Elsevier).

As shown in Figure 4, the expansion of MgO-incorporated concrete increases with the increase in the MgO content and shows a slight reduction after 110 d. Under the sealed condition, concrete with a relatively low content of MgO (ranging from 3% to 5%) significantly shrinks within 28 d. As the age is prolonged, concrete shrinkage is compensated and turns into expansion [26,27]. This phenomenon is due to the fact that the autogenous shrinkage of concrete mostly occurs at the early stage of hydration, whereas the hydration rate of MgO is relatively slow. Thus, the expansion induced by the low addition of MgO is not enough to fully compensate for the shrinkage. In addition, with the increase in the MgO content, the growth rate of concrete expansion becomes higher, and the time to reach a constant strain becomes longer [28,29]. Though the expansion of concrete or mortar increases with the content of MgO, mechanical properties such as compressive strength decrease in the range of 8.8% to 24.7% when using MgO contents of 5% to 20% [30]. The reduction in compressive strength grows with increasing MgO content [7,31,32], and the flexural strength decreases with the addition of MgO as well but reduces less than compressive strength [12,31]. Such reductions relate to the decline of CSH formation, which is caused by less binder content when MgO is introduced [31]. In order to achieve a balance between mechanical properties and shrinkage control, the optimum addition of MgO generally falls between 5% and 8%.

**Figure 5:**
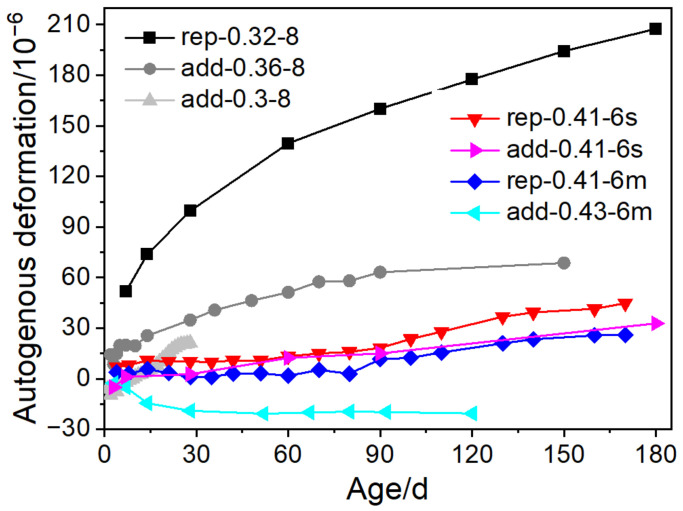
The difference between expansion of concrete with MgO added as replacements (rep) and as additives (add): rep-0.32-8, add-0.36-8, add-0.3-8, rep-0.41-6s, and rep-0.41-6m, add-0.41-6s, and add-0.43-6m (adapted from Ref. [20], 2016, Elsevier; Ref. [28], 2016, *Journal of China Institute of Water Resources and Hydropower Research*; Ref. [35], 2009, *Journal of Wuhan University of Technology*; Ref. [36], 2009, Springer Nature; Ref. [37], 2018, Hu Y.; and Ref. [38], 2020, Thomas Telford Limited).

The addition of MgO is either by partially replacing binders or adding an additive. In Figure 5, concrete with similar w/b ratios and MgO contents is presented to investigate the influence of the manner of MgO addition on deformation. For both the 0.3 and 0.4 w/b ratio groups, concrete with MgO as replacements for binders shows higher expansion than concrete with MgO as additives. When MgO partially replaces binders, the MgO-to-binder ratio in such concrete is higher than that in concrete with MgO as additives (of the same content). Moreover, less cement content in concrete with MgO as replacements gives rise to less autogenous shrinkage than concrete with MgO as additives and the same w/b ratio. With a content of 6%–12% (of the binder mass), the incorporation of MgO can also increase the water demand and reduce the fluidity of cement paste or mortar, regardless of the manner of MgO’s addition [11,33,34]. The fluidity loss aggravates as the content and reactivity of MgO increase. The addition of MgO both increases the specific surface area of the mixture and accordingly shortens the water film thickness and aggravates the agglomeration of particles by altering the zeta potential of the system. The rheology of cementitious materials also influences the microstructure and deformation. Such a relationship still requires further investigation.

### 2.2. Effect of Aggregate and Cementitious Materials

Aggregate not only restrains the deformation of concrete mixed with MgO but also causes changes in the water content of the concrete via water absorption. The expansive strain in concrete specimens is about 10%–30% of the strain in the mortar or paste specimens with the same MgO content (by weight of cement) and w/b ratio [20,26,39,40,41]. The addition of aggregate leads to the fact that the amount of MgO in concrete is lower than that in mortar or paste. Moreover, the elastic modulus of aggregate is higher than that of cement paste, resulting in less deformation under the same expansive stress. Aggregates act as restraints when deformation in cement paste takes place. In addition, the long-term expansion of concrete with MgO and various gradations are listed from the largest to the smallest: first-graded concrete, secondary-graded concrete, and fully graded concrete [42]. As the size of coarse aggregates increases, the restraint effect of the aggregates enhances [43], resulting in less deformation in fully graded concrete. In spite of the restraint effect, the deformation of concrete with MgO is related to water absorption and the linear expansion coefficient of the aggregate. As water absorption by the aggregate increases, the actual w/b ratio of concrete decreases, and free water for MgO hydration decreases, leading to more significant shrinkage and less expansion. As a result, the expansion of concrete with the same MgO content decreases as water absorption by aggregates increases [28,44,45]. 

The expansion of concrete or mortar mixed with MgO increases with the increase in the w/b ratio [28,44,46,47,48,49]. On the one hand, the mechanical strength and elastic modulus of concrete decrease with the increase in the w/b ratio [46]. As a result, under the same expansive stress, the expansion strain of the concrete with a higher w/b ratio is greater. On the other hand, autogenous deformation is the result of the coupled phenomena of the expansion induced by MgO and the self-desiccation of cementitious materials [49], and autogenous shrinkage decreases as the w/b ratio increases. Therefore, concrete with a higher w/b ratio shows higher expansion when the w/b ratio is lower than 0.45. However, when the w/b ratio is above 0.45, the autogenous shrinkage becomes insignificant [50], and the promotion of pore size and porosity by an increment in the w/b ratio becomes evident [51]. Both the volume growth caused by brucite precipitation and the expansive strain induced by crystallization pressure are hardly hindered by such a change in pore structure, and expansion decreases as the w/b ratio of concrete increases.

The type of cement could affect the deformation of concrete with MgO. Concrete that incorporates high-magnesia cement could show certain expansive properties. However, its expansion relates to the calcination temperature of cement and mineral compositions rather than the MgO content [20,44,52,53]. Moreover, with the same w/b ratio and MgO content, the expansion of medium-heat cement concrete under sealed conditions is higher than that of low-heat cement concrete within 28 d and becomes lower at later ages from 56 d to 1085 d [27]. Such phenomena may arise from the fact that the hydration heat evolution and hydration process of medium-heat cement at an early age is faster than that of low-heat cement, which promotes the hydration and expansion of MgO.

**Figure 6:**
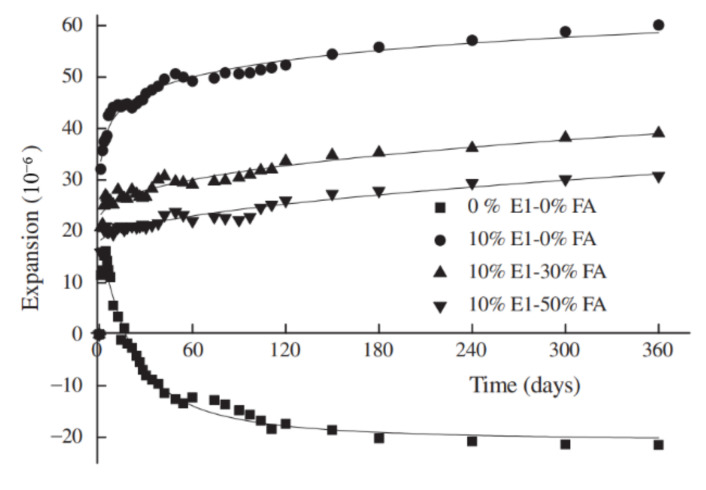
The influence of fly ash content on the autogenous deformation of concrete with MgO (reprinted with permission from Ref. [58], 2013, Elsevier).

Fly ash is a common mineral admixture for concrete that contains MgO. As shown in Figure 6, the expansion of concrete decreases as the fly ash content increases [35,36,54,55,56]. Moreover, according to some studies, with an increase in fly ash content, concrete expansion decreases at early ages but increases at later ages [28]. On the one hand, the pozzolanic reaction of fly ash reduces the alkalinity of the cement matrix and consequently decreases the crystallization pressure of MgO, which exerts a negative impact on the expansive deformation of concrete. On the other hand, the incorporation of fly ash mitigates the autogenous shrinkage of cement paste, especially for those with a relatively low w/b ratio and correspondingly severe autogenous shrinkage, which helps to promote expansive deformation when both shrinkage and expansion take place [57]. The effect of fly ash should be considered from both aspects. Similar to the effect of fly ash, when blast furnace slag and fly ash are both added to the MgO-incorporated concrete with a relatively high w/b ratio of 0.51, the expansion of the concrete decreases with the rising content of the two supplementary cementitious materials [55]. However, when the w/b ratio of the cement paste is sufficient to develop autogenous shrinkage, the addition of slag and fly ash helps to mitigate shrinkage and promote expansion. With the increase in slag addition, the expansion of ternary blended cement paste with MgO hardly changes, while the expansion of ternary blended cement paste decreases with an increase in fly ash content [12,31].

### 2.3. Effect of Curing Conditions

**Figure 7:**
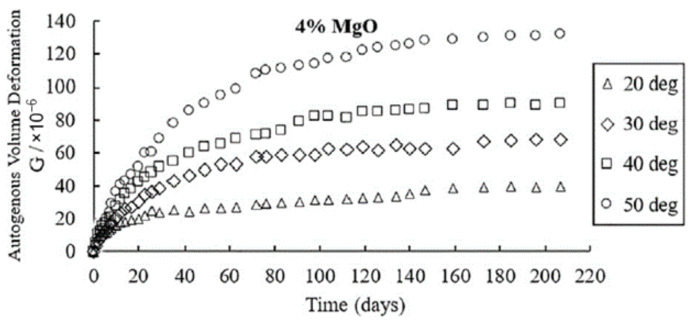
The influence of temperature on the autogenous deformation of concrete mixed with 4% MgO (reprinted with permission from Ref. [62], 2019, Elsevier).

**Figure 8:**
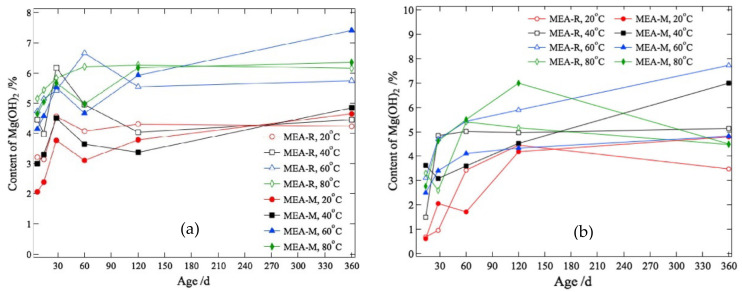
Estimated quantities of Mg(OH)_2_ in cement pastes (6 wt% MgO expansive agent) from 20 ℃ to 80 ℃: (**a**) on the basis of DSC/TG; (**b**) on the basis of QXRD (reprinted with permission from Ref. [32], 2019, Elsevier).

Increasing the curing temperature can promote the expansive behavior of MgO. As shown in Figure 7, as the curing temperature rises, the autogenous expansion of the concrete with MgO increases, the expansive rate accelerates, and the stable age of deformation is reached earlier [54,56,59,60,61,62,63], which indicates the promoting effect of raising the temperature on the hydration of MgO. For high-reactivity MgO, raising the temperature can promote the expansive rate of concrete, but it has no significant effect on the ultimate expansion at later ages (14-30 d). Moreover, the sensitivity of concrete to temperature is lower than that of concrete under sealed conditions [64]. In terms of the temperature history of mass concrete, concrete samples with MgO also undergo thermal shrinkage as temperature decreases, and the temperature sensitivity of concrete is also related to MgO reactivity after deducting temperature deformation from the total autogenous deformation [19]. As shown in Figure 8, the temperature sensitivity increases as reactivity decreases. For medium-activity MgO, the enhanced hydration caused by elevated temperature is more significant than that for high-activity MgO. The hydration rate of MgO with high reactivity is already high enough to reflect the fast expansive rate of cementitious materials. A further increase in hydration rate can cause MgO to hydrate before the final setting of concrete, thus reducing its effective expansion.

Increasing the curing relative humidity (RH) can also promote the expansion of concrete with MgO. The commonly used curing methods for testing include water curing, sealed curing, RH = 60% (dry shrinkage test), RH = 95% (standard curing), etc. When curing humidity of 60% and 100% is compared, the restrained expansion rate of concrete with MgO increases with RH [19]. Comparing water curing and sealed curing, 20 ℃ sealed curing specimens show shrinkage deformation, while water curing specimens show expansion deformation [63]. Comparing water curing and drying at 50% relative humidity, the deformation performance of concrete mixed with MgO also has a similar trend [65]. With the same MgO content, the expansion of concrete under different curing conditions is ranked from the highest to the lowest as follows: water curing, sealed curing, and drying at 60% RH [66]. As shown in Figure 3, compared with a sealed condition, water curing accelerates the hydration of MgO and correspondingly increases the brucite content. Increased curing humidity mitigates the shrinkage of concrete and provides extra water for cement and MgO hydration (water curing), resulting in the promotion of expansion. 

### 2.4. Combination with Other Shrinkage Reduction Methods

Due to its relatively delayed expansion, MgO cannot fully compensate for the early shrinkage of concrete and is often used together with other shrinkage reduction methods. Calcium oxide and calcium sulfoaluminate expansive agents have the characteristics of rapid expansion at early ages [55]. The addition of calcium sulfoaluminate expansive agent can increase the restrained expansion rate of concrete with MgO at early ages (0–28 d) [67]. Additionally, under sealed conditions, the concrete prepared through the low-heat microexpansion of cement and MgO addition is not only compensated for its autogenous shrinkage at the early stage but also shows expansion that steadily increases at the later stage [61,68]. Internal curing by using superabsorbent polymers (SAPs) or other water-reserving materials is often used to mitigate autogenous shrinkage. Under sealed conditions, the addition of SAPs can reduce the early autogenous shrinkage of concrete with MgO and increase later expansion, while under drying conditions, the addition of SAPs can reduce the drying shrinkage and slightly increase the expansion induced by MgO [37]. The extra curing water in SAPs mitigates the drying shrinkage caused by water evaporation and at the same time provides an extra amount of water for MgO hydration. The addition of polypropylene fiber to concrete mixed with MgO can reduce its shrinkage in early ages (0–28 d) and reduce its expansion in later periods (>28 d) [69]. In addition, the types of retarders and water reducers also have a significant impact on the deformation of concrete with MgO under saturated and drying conditions. The combined application of organophosphorus retarders and high-efficiency water-reducing agents results in greater expansion of concrete under water retention conditions, while the addition of naphthalene-based water-reducing agents and sodium gluconate retarders make the drying shrinkage and deformation of concrete larger [70].

## 3. Cracking Resistance

**Figure 9:**
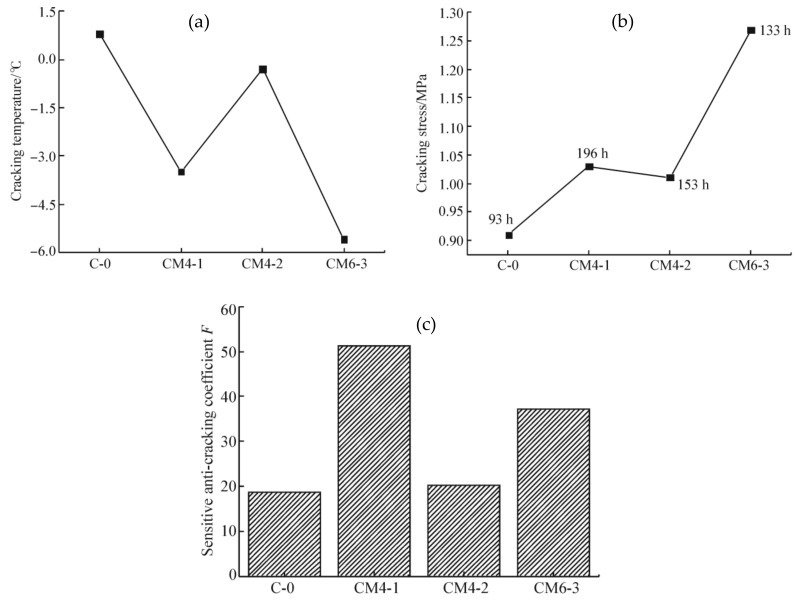
Cracking resistance of MgO incorporated concrete: (**a**) cracking temperature, (**b**) cracking stress, and (**c**) anti-cracking coefficient (reprinted with permission from Ref. [71], 2011, Springer Nature).

The cracking resistance of concrete with MgO is better than ordinary concrete with the same w/b ratio. To evaluate the cracking resistance, tests are carried out under temperature stress, loading, restraint plastic shrinkage, or drying shrinkage, and the common parameters adopted are tensile strength (split or axial), elastic modulus, initial cracking time, cracking temperature, and coefficients that are integrations of these parameters. Lei Wang et al. [22] investigated the effect of reactivity (reaction times of 60 s, 150 s, and 300 s) and dosage (0%, 5%, and 10%) of MgO on the early age cracking resistance of concrete under plastic shrinkage, drying shrinkage, and temperature shrinkage. For plastic shrinkage in 24 h, the initial cracking time shortens, and the cracking resistance decreases as the MgO dosage and reactivity increase, resulting in less bleeding water to mitigate capillary evaporation and ease tensile strength development. For drying shrinkage and temperature stress within 4 d, the concrete cracking resistance obtained from the ring test and TSTM shows growth as the reactivity and dosage of MgO increase, with 10% high-activity MgO (60 s) as the best-performing combination. As mentioned previously, low-activity MgO develops expansion slowly, which leads to almost no shrinkage compensation ability and a decrease in the cracking resistance at early ages. Xia Chen et al. [71,71] used the cracking temperature, tensile strength, and cracking resistance coefficient to characterize the cracking resistance of concrete with MgO under temperature stress, as shown in Figure 9. They found that the incorporation of MgO can effectively reduce the cracking temperature and increase the cracking stress. With a content of 4%, the cracking resistance of concrete with MgO of medium reactivity (109s) is better than that of concrete with high-activity MgO (59s). Hua Li et al. [14] studied the effect of MgO reactivity on the cracking resistance of concrete under temperature stress and found that MgO with low reactivity produces greater expansion in the temperature-dropping stage, and the cracking time of specimens with MgO of relatively high reactivity is delayed by 13.5 days. Consequently, concrete mixed with low-activity MgO (220 s) has better cracking resistance in the early temperature-dropping stage. W. Li et al. [45] studied the influence of aggregate types on the cracking sensitivity of concrete with MgO under temperature stress, using cracking stress, cracking temperature, and temperature shock index as the indicators of cracking resistance performance and found that the cracking resistance of basalt and amphibolite concrete is better than that of limestone and granite concrete. Xu An et al. [72] used the cracking resistance coefficient to characterize the cracking resistance of roller-compacted concrete (RCC) with MgO addition. The cracking resistance coefficient is proportional to ultimate tensile strength, adiabatic temperature rises, and elastic modulus and is inversely proportional to the coefficient of thermal expansion of concrete. With an increase in MgO content, the ultimate tensile strength decreases at 7 d and increases from 28 d to 180 d. The cracking resistance also follows the same pattern, since the expansion of MgO densifies the structure of concrete at later ages. J.A. Forero et al. [30] investigated the fracture toughness of concrete with MgO as a partial substitute for cement at 5%, 10%, and 20% using the wedge splitting test at 28 d. The incorporation of MgO reduces the fracture energy by 13%–53%, and the reduction in fracture energy rises as MgO content is increased. This can be related to the weakening of the interfacial transition zone since the decrease in CSH content and the increase in brucite content both take place. J. Guo et al. [73] studied the effect of MgO in collaboration with a sulfoaluminate expansive agent on the fracture properties of concrete. Though the mixed expansive agent reduced 25% to 35% fracture energy at 14 d and 4% to 12% at 28 d, it promoted the fracture energy by 3% to 8.2% at 60 d. The addition of MgO reduced shrinkage-induced microcracking at 60 d, leading to higher fracture energy at later ages.

## 4. Estimation of Deformation

**Figure 10:**
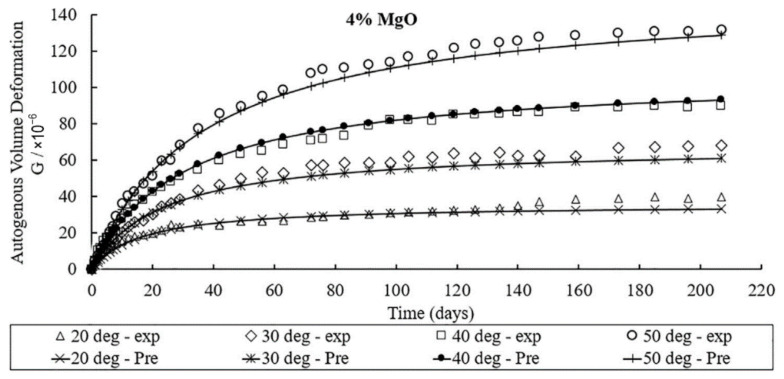
Deformation curve of specimen mixed with 4% MgO content in hyperbolic model under different curing temperature (T) and time (t) conditions (reprinted with permission from Ref. [62], 2019, Elsevier).

The deformation estimation methods for MgO-incorporated concrete mainly include the hyperbolic model, the exponential model, and the tabular interpolation method. In the hyperbolic model, the autogenous deformation of concrete is described as a hyperbolic function of age. As shown in Equation (1) [74], the temperature is introduced as the variable T to cover the influence of curing temperature, and constants a_1_, a_2_, b_1_, and b_2_ are all derived from fitting the function with experimental ultimate autogenous deformation of concrete with MgO cured at different curing temperatures. Due to the nonlinear relationship between deformation and temperature (or age) in Equation (1), the Newton–Raphson method was adopted for calculation. To validate the model, the strain calculated by using the hyperbolic model with temperature history input from the site was compared with the strain directly obtained from the same test points, and the deviation varied from 14% to 33% [75]. In Equation (2), the effects of MgO and fly ash contents on deformation are both considered; the coefficient k_1_ relates to the MgO content, and the coefficient k_2_ relates to the fly ash content [76]. Both k_1_ and k_2_ are determined through regression analysis of the experimental results. As shown in Figure 10, Van Nguyen et al. [62] used a hyperbolic model by considering the combined effects of the MgO content, curing temperature, and age to predict the autogenous deformation of concrete with MgO addition. The predicted deformation results agreed with the measured ones, and the deformation of concrete at different temperatures and MgO contents could be predicted. The exponential model describes the autogenous deformation of concrete as an exponential function of age and temperature, as shown in Equation (3) [77], where *ε_0_* is the ultimate expansion of concrete, and *k*(m) is the coefficient related to the MgO content. Liu et al. [78] introduced the equivalent age as a function of temperature instead of age *τ* in Equation (3) for possible temperature changes as cementitious materials hydrate, especially in mass concrete. When temperature changes in concrete occur, the equivalent age result is more accurate than the ordinary exponential model. Zhang et al. [79] also considered the effect of temperature history and described the expansion rate as being proportional to residual expansion and instantaneous temperature, and the predicted deformation correlates well with the actual deformation. Xu et al. [80] described the strain as an exponential hyperbolic function of the hydration degree of MgO according to the equivalent age, providing the parameters in the model with reasonable physical meanings. The tabular interpolation method uses experimental data on deformation changing with age or temperature to interpolate the strain at certain calculation points during numerical simulation, and no mathematical function of deformation is required [81,82]. A large volume of experimental data with variables such as age, temperature, MgO content, etc., is required as input for regression analysis to obtain the parameters in exponential and hyperbolic models or for interpolation in tabular interpolation methods. Moreover, without sufficient physical meanings for the parameters in these models, it is hard to determine the mechanism of hydration and expansive behavior of MgO in cementitious materials.

**Figure 11:**
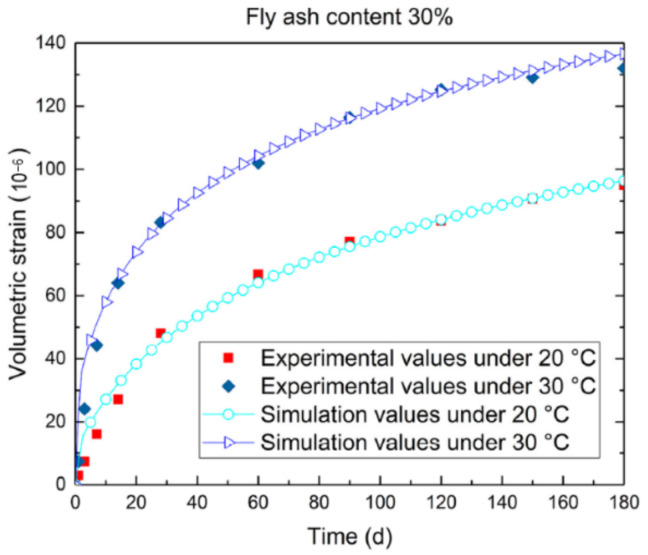
Comparison of the simulated and experimental values of the autogenous volumetric strain of magnesium oxide concrete with various curing temperatures (reprinted with permission from Ref. [84], 2021, *Materials*).

For chemomechanical models, Lecampion et al. [83] used poromechanical equations to predict deformation by incorporating the crystallization pressure of Mg(OH)_2_ in pore space. In Equation (4), the mole balance equation is established assuming that periclase dissolves and brucite forms instantaneously, and the volume change *φ_j_* caused by phase *j* is expressed as the sum of the volume change induced by matrix deformation and the volume of phase *j*. As shown in Equation (5), the volumetric strain is given, and the drained assumption and absent gradient are taken into account during the calculation. Due to the local growth of Mg(OH)_2_ (rather than the preferential growth in large pores) and the underestimation of MgO’s solid–liquid interface energy, the calculated strain value is lower than the experimental result. Feng et al. [84] developed a coupled thermal–chemical–mechanical model for the hydration and deformation of concrete with MEA addition. Under constant temperature, the expansive strain is proportional to the hydration degree of MgO, and heat release and the elastic modulus development are determined by the hydration of cementitious material. In addition, the activation energy of MgO hydration is obtained by using a mathematical derivation method. As shown in Figure 11, the simulated values of autogenous deformation reasonably agree with the experimental values, though slightly higher at early ages. The chemomechanical models of deformation aid in the investigation of the mechanism of expansive behavior of MgO-incorporated concrete.

(1) $$E=\frac{t}{a_{1}T^{a_{2}}t+b_{1}T^{b2}}$$

(2) $$\varepsilon =0.0023k_{1}k_{2}\frac{t}{88.4+10t}$$

(3) $$\varepsilon \left(\tau ,T\right)=k\left(m\right)\varepsilon_{0}\left[1-\exp\left(-aT^{b}\tau^{c}\right)\right]$$

(4) $$n_{j}=\rho_{j}^{o}\varphi_{o}S_{j}+\rho_{j}^{o}(b_{j}\epsilon_{v}+\overset{}{\underset{i}{\sum }}\frac{p_{i}-p_{o}}{N_{ij}}+\frac{\varphi_{o}S_{j}}{k_{j}}\left(p_{j}-p_{o}\right)$$

(5) $$\epsilon_{v}=\frac{bS_{B}\left(p_{B}-p_{o}\right)}{K}$$

## 5. Conclusions

Several conclusions can be drawn from our review of the literature on the deformation and cracking resistance of MgO-incorporated cementitious materials:

(1) The influence of MgO on expansive behavior is classified into three aspects: reactivity, content, and incorporation manner of MgO. Cementitious materials with high-activity MgO (neutralization time less than 100 s) show a higher expansive rate and reach constant deformation earlier, while those with low-activity MgO (neutralization time >200 s) expand slower but show higher expansion at later ages (usually after 60 to 90 days). The difference in the pattern of deformation evolution relates to the hydration process of MgO. The expansion of cementitious materials increases as the MgO content increases, and MgO used as an additive shows higher expansion than MgO used as replacements for binders in concrete.
(2) The effect of concrete matrix on expansive behavior is divided into the w/b ratio, cement composition, and supplementary cementitious materials. When the w/b ratio is lower than 0.45, it promotes the expansion of cementitious materials with MgO, as the increase in the w/b ratio helps to mitigate autogenous shrinkage and provides sufficient free water for MgO hydration. For cementitious materials with a w/b ratio higher than 0.45, expansion decreases as the w/b ratio increases due to the reduced autogenous shrinkage and coarsening of the pore structure. Low-heat cement slows down the hydration process and hydration heat evolution when compared to ordinary and medium-heat cement, resulting in less expansion. With the incorporation of fly ash and slag, the expansion of cementitious material with MgO is reduced, and at the same time, autogenous shrinkage is mitigated. The effect of fly ash and slag has both advantages and disadvantages on deformation, which requires investigation under specific circumstances. In addition, coarse aggregates restrain the concrete matrix to limit deformation and affect the paste through water adsorption.
(3) The expansion of cementitious materials with MgO increases as the curing relative humidity rises, and elevated temperature can significantly improve the expansion, especially for low-activity MgO. Water curing at elevated temperatures is the most effective method for promoting expansion among all the curing conditions.
(4) The cracking resistance of cementitious materials is improved when MgO is added as an expansive agent. The addition of MgO can improve tensile strength and reduce the cracking temperature of cementitious materials during the cooling process.
(5) The methods for estimating the deformation of concrete or paste containing MgO are the hyperbolic model, the exponential model, and the tabular interpolation method. With certain revisions to these methods, such as introducing the equivalent age and activation energy of MgO, the predicted results can reach a reasonable level of accuracy when a considerable number of experimental data have been input. Chemomechanical models, though not thoroughly investigated, are essential for uncovering the relationship between the hydration of MgO and the expansion of cementitious materials.
(6) There are several issues related to the deformation of cementitious materials that are still under debate or have not been explored. Supplementary cementitious materials such as silica fume, glass powder, and other common materials in concrete may alter the chemistry of the matrix and pore solution, which can exert an impact on MgO’s expansive behavior. Though the correlation between the specific surface area (reaction time) of MgO and the dissolution rate has been established, the quantitative relationship between the hydration process of MgO and the expansive deformation of concrete is yet to be determined, as the driving force of expansion is under debate. Furthermore, with the study of MgO hydration in cementitious systems in terms of both the kinetics and thermodynamic aspects and the influence of MgO on the microstructure of the matrix, it would be clear to determine the chemomechanical process during hydration and establish a model for the prediction and optimization of the deformation of MgO-incorporated cementitious materials.
